# Twoline Skiffia's Latency to Exit a Refuge and to Locate Food When Socialising With Invaders and Raising Temperatures

**DOI:** 10.1002/ece3.70813

**Published:** 2025-01-16

**Authors:** Abigail Santiago‐Arellano, Javier Alcocer, Erick de la Barrera, Morelia Camacho‐Cervantes

**Affiliations:** ^1^ Instituto de Ciencias del Mar y Limnología Universidad Nacional Autónoma de México (UNAM), Ciudad Universitaria Mexico City Mexico; ^2^ Posgrado en Ciencias del Mar y Limnología UNAM, Ciudad Universitaria Mexico City Mexico; ^3^ Grupo de Investigación en Limnología Tropical, Facultad de Estudios Superiores Iztacala UNAM Tlalnepantla Mexico; ^4^ Instituto de Investigaciones en Ecosistemas y Sustentabilidad UNAM Morelia Mexico

**Keywords:** behaviour, global warming, goodeids, invasive species, poeciliids, risk taking

## Abstract

Aquatic ecosystems are reservoirs of biodiversity and are highly threatened. Among the main threats to biodiversity are invasive species and global warming, the later has allowed the establishment of invasive species from originally warmer climates outside their native range by reducing the barriers to their establishment and distribution. Behaviour is the immediate response that species modify to counteract changes in their environment. Latency to respond to certain stimuli is an indicator of different behavioural tendencies associated with boldness, for example, quickly leaving a shelter could lead to benefits like finding a mate or locating food faster. We investigated the latency to exit a refuge and to locate food of the native twoline skiffia (*Skiffia bilineata*) from central Mexico at three temperatures (18°C, 23°C and 28°C) and in the presence/absence of invasive guppies ( *Poecilia reticulata* ). Our results are the first to show native fish could benefit from associating with invaders when emerging from a refuge and locating food under higher temperatures, but they would find themselves at the extreme of their thermal tolerance. Evidence of positive outcomes from biological invasions is increasing; however, further research is needed to understand if potential benefits for natives are temporary, which may make biological invasions less detrimental during the initial stages.

## Graphical abstract

Native twoline skiffia's behaviour in response to increasing temperatures and coexistence with invasive guppies was assessed and results uncovered potential benefits for the native fish, including quicker emergence from shelters and more efficient food location when associating with invasive species. However, we emphasise the need for further investigation into the temporary nature of these benefits, as very likely there are long‐term implications of such interactions.

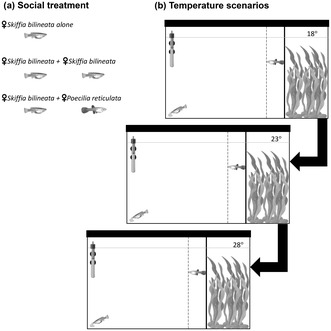

## RESUMEN

Los ecosistemas acuáticos son reservorios de biodiversidad y están altamente amenazados. Una de las principales amenazas para la biodiversidad son las especies invasoras y el calentamiento global que ha permitido el establecimiento de especies invasoras fuera de su rango de distribución nativa al reducir las barreras para su establecimiento y distribución. El comportamiento es la respuesta inmediata que las especies modifican para contrarrestar los cambios en su entorno. La latencia para responder a ciertos estímulos es un indicador de diferentes tendencias de comportamiento asociadas con la audacia; por ejemplo, abandonar rápidamente de un refugio podría resultar en beneficios como encontrar pareja o localizar alimento más rápidamente. Investigamos la latencia para salir de un refugio y localizar alimento del Tiro de dos rayas (*Skiffia bilineata*) nativo del centro de México a tres temperaturas (18°C, 23°C y 28°C) y en presencia o ausencia de guppies invasores ( *Poecilia reticulata* ). Nuestros resultados representan el primer hallazgo de que los peces nativos podrían beneficiarse de asociarse con invasores al salir de un refugio y localizar alimento bajo temperaturas más altas, pero se encontrarían en el extremo de su tolerancia térmica. La evidencia de resultados positivos de invasiones biológicas está aumentando, sin embargo, se necesita más investigación para entender si los beneficios potenciales para los nativos son temporales, lo que podría hacer que las invasiones biológicas sean menos perjudiciales durante las primeras etapas.

## 1 Introduction

Aquatic ecosystems are essential for biodiversity and human welfare because they provide important ecosystem services and have a high rate of endemicity (Green et al. 2015). However, freshwater ecosystems and species are experiencing severe threats (Boteler, Bodle, and Donat 2017). Freshwater ecosystems cover at least 1% of the world's surface and represent 7% of the 1.8 million described species, of which 40% are fishes (Downing 2014; Levêque et al. 2007). Despite their importance to global biodiversity and their vital role in the survival, well‐being and livelihoods of humans, human activities are degrading freshwater ecosystems, leading to an extinction crisis (Lewis 2006). Historically, river–wetland corridors were ubiquitous across vast stretches of alluvial valleys, but their presence has decreased worldwide (due to various human activities and anthropogenic impacts) (Wohl et al. 2021). Furthermore, recent climate change drives other threats to freshwater ecosystems like eutrophication and contamination (Keegan, White, and Macinnis‐Ng 2022).

Biological invasions, which are often caused my human activities, and global warming are increasingly recognised among the main threats to biodiversity, especially in freshwater ecosystems. These processes are often considered independent threats, although they can act synergistically (Kolar and Lodge 2000; Flory, Dillon, and Hiatt 2022). Climate change not only directly impacts natural communities (Urban et al. 2016) but also has the potential to exacerbate the adverse effects of aquatic invasive species through various mechanisms (Malhi et al. 2022). These effects include competition and predation on native species, as well as disease transmission (Rahel and Olden 2008). Additionally, rising temperatures are expected to expand the potential range of warm‐water alien species, either escaping or being released from aquaculture (Lodge et al. 2000; Padilla and Williams 2004). Tropical species inhabit places where environmental temperature remains stable throughout most of the year (Huey et al. 2009), rendering them particularly vulnerable to predictions of global warming as they are suited to temperatures with little variability across seasons (Pedersen et al. 2021).

Human activities impact the environment, influencing population dynamics, species survival and dispersion, as organisms interact with their surroundings (Berger‐Tal and Saltz 2016). In response to these environmental changes, species modify their behaviour as an immediate adaptive strategy (Krause and Ruxton 2002; Parmesan 2006). Environmental changes, particularly those attributed to climate change and invasive species, have significant implications for species behaviour. For example, climate change can facilitate the expansion of the distribution range of invasive species by increasing the potential establishment area, as invasive species typically have greater evolutionary tolerance to temperature changes (Finch et al. 2021). Additionally, global warming affects organisms' metabolism; for instance, in fish, an increase in temperature also increases their metabolic requirements, which could affect their interactions with other species and their activity (Alfonso et al. 2021).

Behavioural traits linked to invasion success include boldness, an increased predisposition to aggression, high activity, exploration and dispersal; these behaviours facilitate population expansion and drive competition with native species (Chapple, Simmonds, and Wong 2012; Pintor, Sih, and Bauer 2008; Rehage and Sih 2004; Tsutsui, Suarez, and Grosberg 2003). In particular, boldness is relevant to the invasion process as it influences dispersal and the search for vital resources (Cote et al. 2010; Monceau et al. 2015). Boldness is the adjustment of an individual's tendencies to take risks in dangerous situations, bolder individuals tend to have high rates of dispersal, exploration, aggression and fast life‐history strategies, which can form a ‘bold‐type’ pace‐of‐life syndrome (Réale et al. 2007).

Successful invasive species often exhibit bold‐type invasion syndromes, characterised by a series of behavioural traits that enhance invasion success at multiple stages of the introduction process. While the bold behaviour of invaders facilitates invasion success, it can also increase susceptibility to predation depending on the context among individuals, populations and species (Rands et al. 2003; Smith and Blumstein 2008). Nevertheless, in dynamic environments, risk‐taking behaviour can improve survival prospects by providing individuals with opportunities to discover new resource options amidst declining quality or availability (Luttbeg and Sih 2010). Indeed, in aquatic systems, elevated temperatures can impact animal personalities, such as boldness, which may subsequently influence the invasive potential of species through enhanced aggression or exploratory behaviours. For example, invasive poikilothermic species, like *Procambarus clarkii*, exhibit increased boldness and aggression as a response to even slight temperature rises, likely as an ecological compensation mechanism for increased metabolic demands (Zhao and Feng 2015). This increase in boldness under warming conditions may support more effective resource acquisition and establishment in new environments. Similarly, research on *Cherax destructor* demonstrates that temperature elevations can increase exploration tendencies, a behavioural syndrome positively correlated with activity and boldness, which may further promote invasion success in warming environments (Ferderer, Davis, and Wong 2022).

The twoline skiffia (*Skiffia bilineata*, syn. *Neotoca bilineata* ) is a fish that is endemic to the Central of Mexico (Fricke, Eschmeyer, and Van der Laan 2018). It is currently in the Endangered category of the IUCN Red List of Threatened Species (Koeck 2019). Twoline skiffias have undergone local extinctions in more than 50% of the sites where they were previously reported (De La Vega‐Salazar, Avila‐Luna, and Macías‐Garcia 2003). The decline of goodeid populations has been partially attributed to the invasion of guppies ( *Poecilia reticulata* ) (Magurran 2009; Gesundheit and Macías Garcia 2018). Twoline skiffias socialise with invasive guppies, which has been found to negatively affect their foraging behaviour (Camacho‐Cervantes, Palomera‐Hernadez, and García 2019) and their reproductive behaviour following sexual harassment by guppies (Valero, Macías Garcia, and Magurran 2008). On the other hand, previous research has found that heterospecific social interactions between invasive guppies and goodeids are beneficial for the guppies. These interactions show that guppies exhibit a greater willingness to explore, acquire information about the invaded area and increase their foraging efficiency (Camacho‐Cervantes et al. 2014; Camacho‐Cervantes, Ojanguren, and Magurran 2015; Santiago‐Arellano, Palomera‐Hernandez, and Camacho‐Cervantes 2021).

Twoline skiffias and guppies share water bodies in Central Mexico. Predictions for Michoacán State, which is within twoline skiffias' native distribution, project annual temperature could increase 1.4°C by 2030, 2.2°C by 2060 and 3.6°C by 2090 (Sáenz‐Romero et al. 2010). Furthermore, the average annual temperature records for a nearby area (Cuitzeo, Michoacán) range from 14°C to 18°C, with extremes of 35°C–40°C down to 0°C–10°C; reporting the lowest values in the highest areas at least once a year (Soto‐Galera et al. 1999). We aimed to investigate how invasive guppies influenced the foraging and risk‐taking behaviour of native twoline skiffia across multiple, ecologically relevant temperature treatments. Our previous studies have tested the benefits that invaders get (via an increase in boldness) from associating with natives but as far as we know the interactions for the natives are disadvantageous by increasing competition (Camacho‐Cervantes, Palomera‐Hernadez, and García 2019; Camacho‐Cervantes et al. 2014; Santiago‐Arellano, Palomera‐Hernandez, and Camacho‐Cervantes 2021). Therefore, we hypothesise that the presence of guppies and warmer temperatures will affect the time it takes for twoline skiffias to exit a refuge and locate food. Given that goodeids are facing both biological invasions and climate change, studying their behaviour is timely and valuable for understanding the mechanisms behind their population declines.

## 2 Materials and Methods

### 2.1 Subject Species

Twoline skiffias inhabit calm, muddy, shallow and commonly slow‐moving freshwater river systems, in depths up to 1 m with dense vegetation (Koeck 2019). This species is among the smaller goodeids. They are sexually dimorphic: females can measure up to 42 mm while males are about 3 mm smaller (Miller and Minckley 2005). The optimal temperature range of the twoline skiffia is from 19°C to 22°C (Ornelas‐Garcia et al. 2012). However, this fish can experience temperatures ranging from 5.9°C to 26.4°C throughout the year (MCC's unpublished data from a water body within their native distribution in Michoacan, Mexico).

The guppy *Poecilia reticulata* is an invasive freshwater fish of the family Poeciliidae with a natural distribution range in Trinidad, Guyana, Venezuela and Suriname (Magurran 2005). Currently, guppies are found on all continents, except Antarctica (Deacon, Ramnarine, and Magurran 2011) and in Mexico, they are found throughout the central plateau (Gesundheit and Macías Garcia 2018). In the wild, guppies inhabit aquatic habitats ranging from highly turbid to pristine ponds, canals or lakes (Lawal, Edokpayi, and Osibona 2012). Guppies are omnivorous and display strong sexual dimorphism; females reach a body length of around 40 mm and up to 60 mm, while males can reach up to 40 mm (Magurran 2005). Their thermal tolerance is wide, and they can survive from 12°C to 39.6°C (Fujio, Nakajima, and Nagahama 1990; Grinder, Bassar, and Auer 2020).

### 2.2 Experimental Design

Experiments were carried out at the Instituto de Ciencias del Mar y Limnología (Universidad Nacional Autonoma de Mexico) from the 3rd of September 2021 to the 28th of March 2022. Twoline skiffias were collected from outdoor ponds in the Instituto de Ecologia, UNAM, and originally collected in the wild in 2000 in Michoacan (19°52′11″ N, 100°58′22″ W), while guppies were collected in Hidalgo (20°30′25″ N, 99°14′44″ W) in 2019. After collection, all fish were carefully transported to the laboratory in 8 L breathable bags containing approximately 20 fish per bag, along with Pentabiocare, salt and zeolite. Pentabiocare is a high‐performance colloidal conditioner with a high concentration of Thiamine (Vitamin B1, also an antineurotic vitamin). This solution has the property of helping fish better withstand stress conditions under the different collection stages, such as transport, capture and during the fish acclimation period to the conditions of a new aquarium. Fish were kept in aquaria for 3 weeks prior to the trials in separate stock tanks (40 L) for each species that contained between 25 and 30 fish per tank. Tanks were set up with aged tap water, using an air pump for 6 days to dechlorinate the water before adding it to the aquarium. Water for the aquaria was treated with Pentabiocare (two drops per litre). Prior to the observations, fish were separated into pairs depending on the social treatment to be tested. Each tank in the storage section of the aquarium had a capacity of 20 L, contained a water purification filter, a water pump, gravel at the bottom, a heater and plastic plants. The experimental photoperiod was kept 12L:12D, using an LED lamp (Microlite 7 W, ~620 lm) for each tank.

For the experiments, we selected 18°C and 23°C to observe twoline skiffias on the edge of their described optimal temperature range (Ornelas‐Garcia et al. 2012), and we chose 28°C as the warming scenario. However, it is important to note that guppies have a broader optimal temperature range (Grinder, Bassar, and Auer 2020). Our temperature range is also coincident with the current prediction for the area, which is an increase of 3.6°C (Sáenz‐Romero et al. 2010). We performed pilot trials to find the upper thermal tolerance of twoline skiffias and found that at 30°C, twoline skiffias struggled (they were hyperventilating and remaining still at the bottom of the tank) thus, we kept the upper‐temperature scenario at 28°C. The aquarium temperature was maintained at 22°C (±1°C), and the stock tanks were visually isolated, individuals used in each trial were kept separated for at least 2 weeks prior to observations to avoid familiarity effects (Griffiths and Magurran 1997). Fish were kept for 1 week at each temperature scenario: 18°C, 23°C and 28°C respectively, before data collection. For our experimental purposes and to reach the lower temperatures, the temperature was reduced by one degree every day until reaching 18°C using a MIDEA air conditioning device. High temperatures were calibrated using heaters (Termojet Ecothermal 50 W Lomas) and were raised one degree each day until the desired temperature was reached. Fish were fed daily with commercial flake food (Biomaa).

**FIGURE 1:**
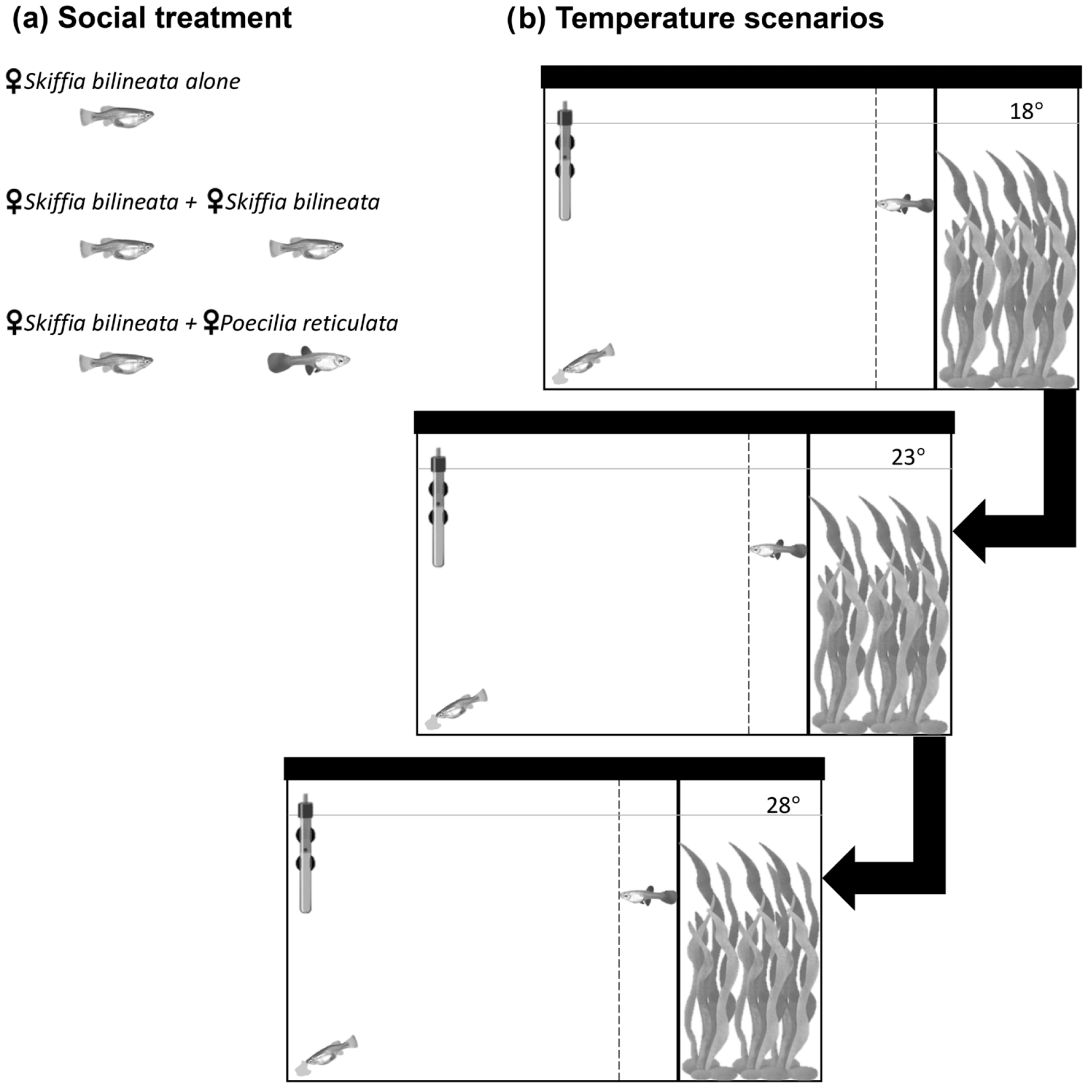
(a) We observed 54 independents focal twoline skiffias under three different social treatments, 18 fish under each treatment. Each focal fish was tested in the temperature scenarios (i.e., repeated measures) as shown in (b) Observation tank diagram. The vertical solid line shows the end of the refuge, and the vertical dotted line shows the distance the fish must cross entirely to be considered out of the refuge area.

For social treatment experiments, we considered three different treatments: (1) focal twoline skiffias alone, (2) focal twoline skiffia accompanied by a conspecific and (3) focal twoline skiffia accompanied by a guppy (Figure 1a), all tested under the three different temperature scenarios. Focal fish were taken from different storage tanks, the temperatures were tested starting at 18°C, then the temperature was increased one degree per day until reaching 23°C. They were kept for a week at this temperature, and they were tested in the same way for the 28°C. To avoid sexually motivated behaviour, only female adult fish were used; being an experiment that focuses on foraging, females are relevant because they spend more time foraging than males (Sievers et al. 2012). Prior to each observation, a commercial food flake of ~2 cm^2^ was placed haphazardly at the bottom of the tank on the area at the opposite side of the refuge area. The experimental observations began when the focal twoline skiffia, and its companion when in paired observations, were gently released into the refuge area. We measured the latency to exit the refuge, defined as the total time required for the focal twoline skiffia to exit the refuge area completely, and the latency to locate food, defined as the time from exiting the refuge until finding the food flake. Fish were considered out of the refuge area once their body was one body length away from the boundary of the refuge (Figure 1). Observations lasted until fish exited the refuge and located food, or up to 15 min for the cases when the focal twoline skiffia did not emerge from the refuge or did not locate food after doing so. We used an observation tank (40 L) made of transparent glass for each tested temperature scenario. Plastic plants were placed to simulate a refuge area on one side of the tank while the food was placed on the opposite side (Figure 1b). The three tanks were identical and were half emptied and refilled with tap water after each observation, ensuring there were no food residues from the previous observation.

A total of 18 independent focal twoline skiffias were observed under each social treatment, and each focal twoline skiffia was observed in the three different temperature scenarios. All observations were made between 9:00 and 13:00 h. To avoid pseudoreplication, pairs for the different social treatments were formed with fish haphazardly selected from five stock tanks of each species (Hurlbert 1984). The sizes of the fish observed ranged from 18 mm to 39 mm. Focal twoline skiffias were size paired with their companion fish for social treatments that included two fish. The difference in size between focal and companion was, on average, 1.9 mm (SD = 1.4 mm).

### 2.3 Statistical Analysis

Differences in the latency of focal twoline skiffias to exit a refuge and locate food afterwards under different temperature scenarios and social treatments were tested using Cox proportional hazards regression model (Cox 1972), a form of survival analysis. To accomplish this, we adapted the R package ‘Survival’ by employing the ‘coxph’ function (Therneau et al. 2000). The HR (Hazard Ratio) value calculated for each predictor variable indicates the magnitude of change in the risk of leaving the shelter associated with different predictor variables, compared to a reference group. Additionally, we used the ‘cluster (ID)’ function to group individuals with the same ID together, and by doing so we incorporated the repeated measures approach in the calculation of standard errors and hypothesis testing. This is crucial for addressing variability associated with different temperature scenarios. Our analytical approach enabled us to evaluate the influence of temperature (18°C, 23°C and 28°C), social treatments (‘alone’, ‘conspecific’ and ‘heterospecific’), and their interaction. Survival curves were generated using the ‘survfit’ function, and the data were visualised using ‘ggplot2’ function. Statistical analyses and plots were constructed in the R software 4.3.1 (R Core Team 2023). We provide the complete code implemented in the Supporting Information.

### 2.4 Ethical Note

The experiments for the purposes of this paper were conducted at the Universidad Nacional Autonoma de Mexico in Mexico City using fish from two species (*Skiffia bilineata* ~ 80 individuals and *Poecilia reticulata* ~ 20 individuals considering focal and partner fish). The experimental design involved behavioural observations in glass aquarium tanks, which did not include any surgery, anaesthesia or other invasive procedure that could have produced distress in the fish. Mortality was negligible (< 5%) and once the experiments were completed, fish were returned to the aquariums to be kept for future research projects.

All methods above were revised and approved by the Subcomité de Bioética de la Comisión de Ética Académica y Responsabilidad Científica de la Facultad de Ciencias, UNAM with the folio: PI_2022_03_05_Santiago; and are in accordance with the Guide for the Care and Use of Laboratory Animals (National Research Council 2011). Fish were transported to the laboratory following the Official Mexican Norm NOM‐051‐ZOO‐1995 for humanitarian treatment in the mobilisation of animals. Laboratory protocols followed all guidelines provided by the Mexican Official Norm NOM‐062‐ZOO‐1999 for the use and maintenance of vertebrates for research purposes.

## 3 Results

### 3.1 Latency to Emerge

**FIGURE 2:**
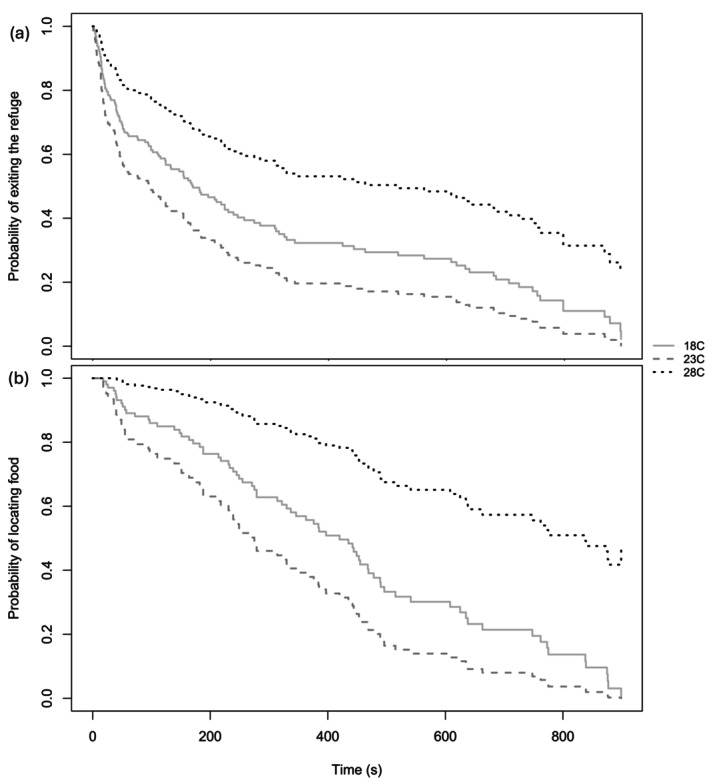
Probability of fish (a) exiting the refuge and (b) locating food. In both panels, solid lines represent 18°C, dashed lines represent 23°C and dots represent 28°C.

Social treatments did not significantly affect the time it took for the focal fish to exit the refuge (HR = 1.438 ± 0.667, *p* = 0.112). Similarly, size did not have a significant effect on the time it took focal fish to leave the refuge (HR = 0.979 ± 0.046, *p* = 0.376). Regarding the effect of temperature, at 28°C focal fish left the refuge significantly faster (HR = 1.644 ± 0.743, *p* = 0.026; Figure 2a). We did not find a significant interaction between temperature and social condition. Nevertheless, at 28°C we found a nonsignificant trend for fish exiting faster when with a heterospecific (HR = 2.315 ± 2.525, *p* = 0.081) than when they were with a conspecific (HR = 1.378 ± 1.518, *p* = 0.508). Twoline skiffias exited the refuge faster at 28°C compared to 18°C, with no significant differences observed between 18°C and 23°C, or between 23°C and 28°C. The model's concordance index was 0.583, indicating moderate predictive accuracy. Number of focal fish that exited the refuge relative to the total number of fish observed are shown in Table 1.

**TABLE 1:** Number of focal fish that exited the refuge and located food (numerator), presented relative to the total number of fish tested in each treatment (denominator). Twoline skiffias are represented in grey and guppies in white.

	Treatment	18°C	23°C	28°C
Exited the refuge	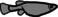	10/18	15/18	17/18
	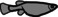 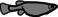	16/18	16/18	18/18
	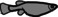 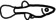	14/18	18/18	17/18
Located food	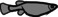	4/10	6/15	8/17
	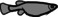 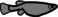	3/16	5/16	11/18
	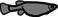 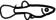	6/14	14/18	14/17

### 3.2 Latency to Locate Food

Social treatments did not significantly affect the time it took for the focal fish to locate food (HR = 0.951 ± 0.502, *p* = 0.860). Size did not have an effect on the time it took focal fish to locate the food once out of the refuge (HR = 1.013 ± 0.057, *p* = 0.570). Regarding the effect of temperature, at 28°C focal fish located food faster than at 18°C and 23°C (HR = 1.951 ± 1.321, *p* = 0.044; Figure 2b). We did not find a significant interaction between temperature and social condition. Nevertheless, we found a nonsignificant trend for fish to locate food faster at 28°C when with a heterospecific (HR = 3.980 ± 1.63, *p* = 0.098) compared to when they were with a conspecific (HR = 0.995 ± 1.64, *p* = 0.996). Twoline Skiffias located food faster at higher temperatures. The model's concordance index was 0.574, indicating fair predictive accuracy. Number of focal fish that located food relative to the total fish that excited the refuge are shown in Table 1.

## 4 Discussion

Twoline skiffias' use of refuge behaviour changed at different temperature scenarios, they had a higher probability of exiting a refuge at 28°C than at 18°C, whether alone or with another fish (conspecific or heterospecific). This behaviour aligns with findings in other fish species, where lower temperatures often correspond with reduced metabolic rates, leading fish to conserve energy by reducing movement and seeking shelter (Cooper, Adriaenssens, and Killen 2018). However, while some fish can detect environmental changes of 1.5°C (Krause, Staaks, and Mehner 1998), twoline skiffias presented a change in their response behaviour, both in latency to exit the refuge and to locate food, only between 18°C and 28°C. The lack of significant behavioural differences between 18°C and 23°C or between 23°C and 28°C suggests that 23°C might represent a moderate zone where neither pronounced energy conservation (seen at 18°C) nor increased activity (observed at 28°C) is necessary. In this range, we hypothesise that twoline skiffias may be balancing metabolic stability with moderate activity levels, thereby reducing the need for extreme refuge use or frequent excursions to search for resources. This aligns with the concept of an optimal thermal zone where activity and metabolic demands are balanced, a phenomenon observed in various species when temperatures do not reach critical upper or lower limits (Frechette et al. 2018). However, an experimental study would be necessary to test this hypothesis and confirm whether such a balance truly influences their behavioural response at moderate temperatures.

As temperatures approach the upper end of an organism's thermal tolerance, there is often a corresponding increase in activity levels to support heightened metabolic functions (Dugdale et al. 2016). In our experiment, as temperatures were raised to 28°C, it is likely that metabolic demand and energy requirement increased, potentially leading to twoline skiffias exiting the refuge faster (Clarke and Johnston 1999). Twoline skiffias presented a significant change in their refuge use behaviour in temperatures 10°C apart, while for the only other goodeid species (*Girardinichthys multiradiatus*) where this has been tested behavioural differences were found in temperatures 4°C apart (Ramírez Carrillo and Macías Garcia 2015). *Girardinichthys multiradiatus* struggles at 28°C, in the present study we found twoline skiffias struggles at 30°C, and on the other hand, guppies' critical maximum temperature was found at 39.6°C (Grinder, Bassar, and Auer 2020), which gives guppies a greater range of adaptation to changing global warming scenarios. If the current warming scenario in twoline skiffias distribution range remains (Sáenz‐Romero et al. 2010), goodeids will be able to survive but they would be close to their upper thermal limit which could cause a change in their biological activity and growth, potentially decreasing their abundance (Jain, Sharma, and Mathur 2013). However, we cannot know for sure if they will survive these predicted climate scenarios. To the best of our knowledge, due to their wide thermal tolerance, increasing temperature will not be enough to prevent guppies from carrying out activities necessary for survival. For example, guppies did not alter their refuge use or swimming activity at 30°C, while skiffias increased their time in a refuge and decreased their swimming activity with rising temperatures (Gomez‐Maldonado and Camacho‐Cervantes 2022).

The thermal tolerance and plasticity of invasive species tend to be higher than that of natives (Devin and Beisel 2007; Vilizzi et al. 2021). An experiment carried out with the invasive mosquitofish ( *Gambusia holbrooki* ), and the endemic Iberian toothcarp ( *Aphanius iberus* ) demonstrated that at higher temperatures, invasive mosquitofish are better at competitively displacing natives through aggression and more efficient foraging (Carmona‐Catot, Magellan, and García‐Berthou 2013). Similarly, *G. multiraduatus* populations in the Zempoala lagoons in Mexico have shifted their breeding season to the colder months after being confronted with rising temperatures and twospot livebearers' ( *Pseudoxiphophorus bimaculatus* ) invasion (Ramírez Carrillo and Macías Garcia 2015). In these two lagoons, temperature increases and biological invasions act synergically. The synergy between invasive species and global warming is known as ‘thermal squeeze’, the displacement of native fauna to restricted areas or seasons where/when temperature is within their thermal ranges (Walker, Monks, and Innes 2019). This has been recognised to happen in other taxa, for example, the increase in winter temperatures in Aotearoa, New Zealand, has affected native mountain beech (*Fuscospora cliffortioides*) while allowing the ship rats ( *Rattus rattus* ) to invade areas that were previously too cold for them (Harris et al. 2022). Nevertheless, contrary to our hypothesis, native twoline skiffias could be deriving benefits from a higher tendency to risk‐taking behaviours by exiting more frequently as the temperature increases. This behaviour is similar to that reported for the Siamese fighting fish ( *Betta splendens* ), who became bolder (i.e., exited a refuge faster and became more active in general) at higher temperatures (Forsatkar et al. 2016). Furthermore, this benefit was maintained even when twoline skiffias were accompanied by invasive guppies. Taken together, we could hypothesise that twoline skiffia exhibit increased overall ‘boldness’ with increasing temperature and invader presence. However, this initial benefit may not be maintained in the long term or under natural conditions, so a study incorporating natural conditions for longer periods could contribute to better understand our results.

Our results suggest that twoline skiffia may find food more frequently at higher temperatures, potentially due to increased metabolic demand. Similarly, Atlantic cod have been observed to modify their size and reproduction period, with a corresponding increase in feeding activity, following global warming scenarios projecting temperature increases of 2°C to 7°C above current levels (Holt and Jørgensen 2014). Additionally, a study carried out on Canadian cod stocks indicate a higher and increasing natural mortality rate associated with rising temperatures (Swain 2011). However, as mentioned above, the long‐term effects of the changes we found on twoline skiffias remain uncertain.

The study of the shy‐bold continuum has focused on the positive balance of the trade‐off between gains and losses, such as foraging or mating opportunities versus predation risk (Ólafsdóttir and Magellan 2016). Variation in boldness is driven by this balance and, as such, is affected by other factors, such as metabolic rate (Krause, Staaks, and Mehner 1998), food deprivation (Godin and Smith 1988) and perception of predation risk (Coleman and Wilson 1998). Most studies on biological invasions focus on the adverse outcomes for native species (Vimercati et al. 2020). However, little is known about the positive effects that could result from the presence of the invasive species (Rodriguez 2006; Braga et al. 2018; Hernández‐Brito et al. 2020). A review carried out by Vimercati et al. (2020) found that, although the evidence for negative impacts is higher, evidence for beneficial impacts is mounting. For example, the monk parakeet (*Myiopsitta monachus*) is the only invasive parrot that builds communal nests, resulting in a collective defence between natives and invaders against predators (Hernández‐Brito et al. 2020, 2021). Our study is the first to find that native fish could benefit from a temperature increase and associations with invaders, but further research is needed to understand if potential benefits for natives are temporary, which may make biological invasions less evident during the initial stages and delay their management.

## Author Contributions

**Abigail Santiago‐Arellano:** conceptualization (equal), data curation (equal), formal analysis (equal), investigation (equal), methodology (equal), writing – original draft (equal), writing – review and editing (equal). **Javier Alcocer:** investigation (equal), supervision (equal), visualization (equal), writing – review and editing (equal). **Erick de la Barrera:** investigation (equal), supervision (equal), visualization (equal), writing – review and editing (equal). **Morelia Camacho‐Cervantes:** conceptualization (equal), data curation (equal), formal analysis (equal), funding acquisition (equal), investigation (equal), methodology (equal), project administration (equal), resources (equal), supervision (equal), validation (equal), visualization (equal), writing – review and editing (equal).

## Conflicts of Interest

The authors declare no conflicts of interest.

## Supporting information


Data S1.

## Data Availability

We attached data and analysis script as Supporting Information (SantiagoArellanoetal2022.R, SantiagoArellanoetal2022Cox.txt).

## References

[ece370813-bib-0001] Alfonso, S. , S. Alfonso , M. Gesto , and B. Sadoul . 2021. “Temperature Increase and Its Effects on Fish Stress Physiology in the Context of Global Warming.” Journal of Fish Biology 98: 1496–1508. 33111333 10.1111/jfb.14599

[ece370813-bib-0002] Berger‐Tal, O. , and D. Saltz . 2016. Conservation Behavior: Applying Behavioral Ecology to Wildlife Conservation and Management, 21. Cambridge, UK: Cambridge University Press.

[ece370813-bib-0003] Boteler, B. S. , R. Bodle , and L. Donat . 2017. The UN Ocean Conference‐June 2017, Guidance to the ENVI Committee of the European Parliament. Brussels, Belgium: European Parliament.

[ece370813-bib-0004] Braga, R. R. , L. Gómez Aparicio , T. Heger , J. R. S. Vitule , and J. M. Jeschke . 2018. “Invasional Meltdown Hypothesis.” In Invasion Biology: Hypotheses and Evidence, 79–91. Wallingford, UK: CAB International.

[ece370813-bib-0005] Camacho‐Cervantes, M. , A. F. Ojanguren , A. E. Deacon , I. W. Ramnarine , and A. E. Magurran . 2014. “Association Tendency and Preference for Heterospecifics in an Invasive Species.” Behaviour 151: 769–780.

[ece370813-bib-0006] Camacho‐Cervantes, M. , A. F. Ojanguren , and A. E. Magurran . 2015. “Exploratory Behaviour and Transmission of Information Between the Invasive Guppy and Native Mexican Topminnows.” Animal Behaviour 106: 115–120.

[ece370813-bib-0007] Camacho‐Cervantes, M. , V. Palomera‐Hernadez , and C. M. García . 2019. “Foraging Behaviour of a Native Topminnow When Shoaling With Invaders.” Aquatic Invasions 14: 3–501.

[ece370813-bib-0008] Carmona‐Catot, G. , K. Magellan , and E. García‐Berthou . 2013. “Temperature‐Specific Competition Between Invasive Mosquitofish and an Endangered Cyprinodontid Fish.” PLoS One 8: e54734. 23382951 10.1371/journal.pone.0054734 PMC3555637

[ece370813-bib-0009] Chapple, D. G. , S. M. Simmonds , and B. B. Wong . 2012. “Can Behavioral and Personality Traits Influence the Success of Unintentional Species Introductions?” Trends in Ecology & Evolution 27: 57–64. 22001529 10.1016/j.tree.2011.09.010

[ece370813-bib-0010] Clarke, A. , and N. M. Johnston . 1999. “Scaling of Metabolic Rate With Body Mass and Temperature in Teleost Fish.” Journal of Animal Ecology 68, no. 5: 893–905. 10.1111/j.1365-2656.2010.01672.x 20180875

[ece370813-bib-0011] Coleman, K. , and D. S. Wilson . 1998. “Shyness and Boldness in Pumpkinseed Sunfish: Individual Differences Are Context‐Specific.” Animal Behaviour 56: 927–936. 9790704 10.1006/anbe.1998.0852

[ece370813-bib-0012] Cooper, B. , B. Adriaenssens , and S. S. Killen . 2018. “Individual Variation in the Compromise Between Social Group Membership and Exposure to Preferred Temperatures.” Proceedings of the Royal Society B: Biological Sciences 285, no. 1880: 20180884. 10.1098/rspb.2018.0884 PMC6015869 29899078

[ece370813-bib-0013] Cote, J. , S. Fogarty , K. Weinersmith , T. Brodin , and A. Sih . 2010. “Personality Traits and Dispersal Tendency in the Invasive Mosquitofish ( *Gambusia affinis* ).” Proceedings of the Royal Society B: Biological Sciences 277, no. 1687: 1571–1579. 10.1098/rspb.2009.2128 PMC2871838 20071380

[ece370813-bib-0014] Cox, D. R. 1972. “Regression Models and Life‐Tables.” Journal of the Royal Statistical Society: Series B: Methodological 34: 187–202.

[ece370813-bib-0015] De La Vega‐Salazar, M. Y. , E. Avila‐Luna , and C. Macías‐Garcia . 2003. “Ecological Evaluation of Local Extinction: The Case of Two Genera of Endemic Mexican Fish, *Zoogoneticus* and *Skiffia* .” Biodiversity and Conservation 12: 2043–2056.

[ece370813-bib-0016] Deacon, A. E. , I. W. Ramnarine , and A. E. Magurran . 2011. “How Reproductive Ecology Contributes to the Spread of a Globally Invasive Fish.” PLoS One 6: e24416. 21957449 10.1371/journal.pone.0024416 PMC3176282

[ece370813-bib-0017] Devin, S. , and J.‐N. Beisel . 2007. “Biological and Ecological Characteristics of Invasive Species: A Gammarid Study.” Biological Invasions 9: 13–24.

[ece370813-bib-0018] Downing, J. A. 2014. “Productivity of Freshwater Ecosystems and Climate Change.” In Global Environmental Change, edited by B. Freedman , 221–229. the Netherlands: Springer.

[ece370813-bib-0019] Dugdale, S. J. , J. Franssen , E. Corey , N. E. Bergeron , M. Lapointe , and R. A. Cunjak . 2016. “Main Stem Movement of Atlantic Salmon Parr in Response to High River Temperature.” Ecology of Freshwater Fish 25, no. 3: 429–445.

[ece370813-bib-0020] Ferderer, A. , A. R. Davis , and M. Y. L. Wong . 2022. “Temperature and Body Size Influence Personality and Behavioural Syndromes in an Invasive Crayfish.” Animal Behaviour 190: 187–198.

[ece370813-bib-0021] Finch, D. M. , J. L. Butler , J. B. Runyon , et al. 2021. “Effects of Climate Change on Invasive Species.” In Invasive Species in Forests and Rangelands of the United States: A Comprehensive Science Synthesis for the United States Forest Sector, 57–83. Berlin/Heidelberg, Germany: Springer.

[ece370813-bib-0022] Flory, S. L. , W. Dillon , and D. Hiatt . 2022. “Interacting Global Change Drivers Suppress a Foundation Tree Species.” Ecology Letters 25: 971–980. 35132744 10.1111/ele.13974

[ece370813-bib-0023] Forsatkar, M. N. , M. A. Nematollahi , P. A. Biro , and C. Beckmann . 2016. “Individual Boldness Traits Influenced by Temperature in Male Siamese Fighting Fish.” Physiology & Behavior 165: 267–272. 27520588 10.1016/j.physbeh.2016.08.007

[ece370813-bib-0024] Frechette, D. M. , S. J. Dugdale , J. J. Dodson , and N. E. Bergeron . 2018. “Understanding Summertime Thermal Refuge Use by Adult Atlantic Salmon Using Remote Sensing, River Temperature Monitoring, and Acoustic Telemetry.” Canadian Journal of Fisheries and Aquatic Sciences 75, no. 11: 1999–2010.

[ece370813-bib-0025] Fricke, R. , W. N. Eschmeyer , and R. Van der Laan . 2018. Catalog of Fishes: Genera, Species, References. San Francisco, CA, USA: California Academy of Sciences. http://researcharchive.calacademy.org/research/ichthyology/catalog/fishcatmain.Asp.

[ece370813-bib-0026] Fujio, Y. , M. Nakajima , and Y. Nagahama . 1990. “Detection of a Low Temperature‐Resistant Gene in the Guppy ( *Poecilia reticulata* ), With Reference to Sex‐Linked Inheritance.” Japanese Journal of Genetics 65: 201–207.

[ece370813-bib-0027] Gesundheit, P. , and C. Macías Garcia . 2018. “The Role of Introduced Species in the Decline of a Highly Endemic Fish Fauna in Central Mexico.” Aquatic Conservation: Marine and Freshwater Ecosystems 28: 1384–1395.

[ece370813-bib-0028] Godin, J.‐G. J. , and S. A. Smith . 1988. “A Fitness Cost of Foraging in the Guppy.” Nature 333: 69–71.

[ece370813-bib-0029] Gomez‐Maldonado, S. , and M. Camacho‐Cervantes . 2022. “Effect of a Temperature Gradient on the Behaviour of an Endangered Mexican Topminnow and an Invasive Freshwater Fish.” Scientific Reports 12: 20584. 36446867 10.1038/s41598-022-24755-9 PMC9709034

[ece370813-bib-0030] Green, P. A. , C. J. Vörösmarty , I. Harrison , T. Farrell , L. Sáenz , and B. M. Fekete . 2015. “Freshwater Ecosystem Services Supporting Humans: Pivoting From Water Crisis to Water Solutions.” Global Environmental Change 34: 108–118.

[ece370813-bib-0031] Griffiths, S. W. , and A. E. Magurran . 1997. “Familiarity in Schooling Fish: How Long Does It Take to Acquire?” Animal Behaviour 53: 945–949.

[ece370813-bib-0032] Grinder, R. M. , R. D. Bassar , and S. K. Auer . 2020. “Upper Thermal Limits Are Repeatable in Trinidadian Guppies.” Journal of Thermal Biology 90: 102597. 32479392 10.1016/j.jtherbio.2020.102597

[ece370813-bib-0033] Harris, H. A. , D. Kelly , J. Innes , and R. B. Allen . 2022. “Invasive Species and Thermal Squeeze: Distribution of Two Invasive Predators and Drivers of Ship Rat ( *Rattus rattus* ) Invasion in Mid‐Elevation Fuscospora Forest.” Biological Invasions 24, no. 8: 2547–2559.

[ece370813-bib-0034] Hernández‐Brito, D. , G. Blanco , J. L. Tella , and M. Carrete . 2020. “A Protective Nesting Association With Native Species Counteracts Biotic Resistance for the Spread of an Invasive Parakeet From Urban Into Rural Habitats.” Frontiers in Zoology 17: 13. 32411270 10.1186/s12983-020-00360-2 PMC7206781

[ece370813-bib-0035] Hernández‐Brito, D. , M. Carrete , G. Blanco , et al. 2021. “The Role of Monk Parakeets as Nest‐Site Facilitators in Their Native and Invaded Areas.” Biology 10: 683. 34356538 10.3390/biology10070683 PMC8301312

[ece370813-bib-0036] Holt, R. E. , and C. Jørgensen . 2014. “Climate Warming Causes Life‐History Evolution in a Model for Atlantic Cod ( *Gadus morhua* ). *Conservation* .” Physiology 2: cou050. 10.1093/conphys/cou050 PMC4806736 27293671

[ece370813-bib-0037] Huey, R. B. , C. A. Deutsch , J. J. Tewksbury , et al. 2009. “Why Tropical Forest Lizards Are Vulnerable to Climate Warming.” Proceedings of the Royal Society B: Biological Sciences 276: 1939–1948. 10.1098/rspb.2008.1957 PMC2677251 19324762

[ece370813-bib-0038] Hurlbert, S. H. 1984. “Pseudoreplication and the Design of Ecological Field Experiments.” Ecological Monographs 54: 187–211.

[ece370813-bib-0039] Jain, S. , G. Sharma , and Y. P. Mathur . 2013. “Effects of Temperature Variations on Fish in Lakes.” International Journal of Engineering Research & Technology 2: 2516–2523.

[ece370813-bib-0040] Keegan, L. J. , R. S. White , and C. Macinnis‐Ng . 2022. “Current Knowledge and Potential Impacts of Climate Change on New Zealand's Biological Heritage.” New Zealand Journal of Ecology 46: 3467.

[ece370813-bib-0041] Koeck, M. 2019. “ *Neotoca bilineata* .” In The IUCN Red List of Threatened Species. Gland, Switzerland: IUCN. 10.2305/IUCN.UK.2019-2.RLTS.T191713A2000012.en.

[ece370813-bib-0042] Kolar, C. S. , and D. M. Lodge . 2000. “Freshwater Nonindigenous Species: Interactions With Other Global Changes.” In Invasive Species in a Changing World, edited by H. A. Mooney and R. J. Hobss , 3–30. Washington DC: Island Press.

[ece370813-bib-0043] Krause, J. , and G. D. Ruxton . 2002. Living in Groups. Oxford, UK: Oxford University Press.

[ece370813-bib-0044] Krause, J. , G. Staaks , and T. Mehner . 1998. “Habitat Choice in Shoals of Roach as a Function of Water Temperature and Feeding Rate.” Journal of Fish Biology 53: 377–386.

[ece370813-bib-0045] Lawal, M. , C. A. Edokpayi , and A. O. Osibona . 2012. “Food and Feeding Habits of the Guppy, *Poecilia reticulata* , From Drainage Canal Systems in Lagos, Southwestern Nigeria.” West Africa Journal of Applied Ecology 20: 1–9.

[ece370813-bib-0046] Levêque, C. , T. Oberdorff , D. Paugy , M. L. J. Stiassny , and P. A. Tedesco . 2007. “Global Diversity of Fish (Pisces) in Freshwater.” Hydrobiologia 595: 545–567.

[ece370813-bib-0047] Lewis, O. T. 2006. “Climate Change, Species–Area Curves and the Extinction Crisis.” Philosophical Transactions of the Royal Society, B: Biological Sciences 361: 163–171. 10.1098/rstb.2005.1712 PMC1831839 16553315

[ece370813-bib-0048] Lodge, D. M. , C. A. Taylor , D. M. Holdich , and J. Skurdal . 2000. “Nonindigenous Crayfishes Threaten North American Freshwater Biodiversity: Lessons From Europe.” Fisheries 25: 7–20.

[ece370813-bib-0049] Luttbeg, B. , and A. Sih . 2010. “Risk, Resources and State‐Dependent Adaptive Behavioural Syndromes.” Philosophical Transactions of the Royal Society, B: Biological Sciences 365: 3977–3990. 10.1098/rstb.2010.0207 PMC2992746 21078650

[ece370813-bib-0050] Magurran, A. E. 2005. Evolutionary Ecology: The Trinidadian Guppy. New York, USA: Oxford University Press.

[ece370813-bib-0051] Magurran, A. E. 2009. “Threats to Freshwater Fish.” Science 325: 1215–1216. 19729647 10.1126/science.1177215

[ece370813-bib-0052] Malhi, Y. , T. Lander , E. le Roux , et al. 2022. “The Role of Large Wild Animals in Climate Change Mitigation and Adaptation.” Current Biology 32: R181–R196. 35231416 10.1016/j.cub.2022.01.041

[ece370813-bib-0053] Miller, R. R. , and W. L. Minckley . 2005. Freshwater Fishes of Mexico. Chicago: University of Chicago Press.

[ece370813-bib-0054] Monceau, K. , J. Moreau , J. Poidatz , O. Bonnard , and D. Thiéry . 2015. “Behavioral Syndrome in a Native and an Invasive Hymenoptera Species.” Insect Science 22, no. 4: 541–548. 24831877 10.1111/1744-7917.12140

[ece370813-bib-0055] National Research Council . 2011. Guide for the Care and Use of Laboratory Animals. 8th ed. Washington, DC: National Academies Press.

[ece370813-bib-0056] Ólafsdóttir, G. Á. , and K. Magellan . 2016. “Interactions Between Boldness, Foraging Performance and Behavioural Plasticity Across Social Contexts.” Behavioral Ecology and Sociobiology 70: 1879–1889. 27784956 10.1007/s00265-016-2193-0 PMC5054052

[ece370813-bib-0057] Ornelas‐Garcia, C. P. , F. Alda , E. Díaz‐Pardo , A. Gutiérrez‐Hernández , and I. Doadrio . 2012. “Genetic Diversity Shaped by Historical and Recent Factors in the Live‐Bearing Twoline Skiffia *Neotoca bilineata* .” Journal of Fish Biology 81: 1963–1984. 23130693 10.1111/j.1095-8649.2012.03456.x

[ece370813-bib-0058] Padilla, D. K. , and S. L. Williams . 2004. “Beyond Ballast Water: Aquarium and Ornamental Trades as Sources of Invasive Species in Aquatic Ecosystems.” Frontiers in Ecology and the Environment 2: 131–138.

[ece370813-bib-0059] Parmesan, C. 2006. “Ecological and Evolutionary Responses to Recent Climate Change.” Annual Review of Ecology, Evolution, and Systematics 37: 637–669.

[ece370813-bib-0060] Pedersen, J. S. T. , F. D. Santos , D. van Vuuren , et al. 2021. “An Assessment of the Performance of Scenarios Against Historical Global Emissions for IPCC Reports.” Global Environmental Change 66: 102199.

[ece370813-bib-0061] Pintor, L. M. , A. Sih , and M. L. Bauer . 2008. “Differences in Aggression, Activity and Boldness Between Native and Introduced Populations of an Invasive Crayfish.” Oikos 117: 1629–1636.

[ece370813-bib-0062] R Core Team . 2023. R: A Language and Environment for Statistical Computing. Vienna, Austria: R Foundation for Statistical Computing.

[ece370813-bib-0063] Rahel, F. J. , and J. D. Olden . 2008. “Assessing the Effects of Climate Change on Aquatic Invasive Species.” Conservation Biology 22: 521–533. 18577081 10.1111/j.1523-1739.2008.00950.x

[ece370813-bib-0064] Ramírez Carrillo, E. , and C. Macías Garcia . 2015. “Limited Options for Native Goodeid Fish Simultaneously Confronted to Climate Change and Biological Invasions.” Biological Invasions 17: 245–256.

[ece370813-bib-0065] Rands, S. A. , G. Cowlishaw , R. A. Pettifor , J. M. Rowcliffe , and R. A. Johnstone . 2003. “Spontaneous Emergence of Leaders and Followers in Foraging Pairs.” Nature 423: 432–434. 12761547 10.1038/nature01630

[ece370813-bib-0066] Réale, D. , S. M. Reader , D. Sol , P. T. McDougall , and N. J. Dingemanse . 2007. “Integrating Animal Temperament Within Ecology and Evolution.” Biological Reviews 82: 291–318. 17437562 10.1111/j.1469-185X.2007.00010.x

[ece370813-bib-0067] Rehage, J. S. , and A. Sih . 2004. “Dispersal Behavior, Boldness, and the Link to Invasiveness: A Comparison of Four *Gambusia* Species.” Biological Invasions 6: 379–391.

[ece370813-bib-0068] Rodriguez, L. F. 2006. “Can Invasive Species Facilitate Native Species? Evidence of How, When, and Why These Impacts Occur.” Biological Invasions 8: 927–939.

[ece370813-bib-0069] Sáenz‐Romero, C. , G. E. Rehfeldt , N. L. Crookston , et al. 2010. “Spline Models of Contemporary, 2030, 2060 and 2090 Climates for Mexico and Their Use in Understanding Climate‐Change Impacts on the Vegetation.” Climatic Change 102: 595–623.

[ece370813-bib-0070] Santiago‐Arellano, A. , V. Palomera‐Hernandez , and M. Camacho‐Cervantes . 2021. “Con‐ and Heterospecific Shoaling Makes Invasive Guppies More Risk Taking.” Frontiers in Ecology and Evolution 9: 156.

[ece370813-bib-0071] Sievers, C. , E. M. Willing , M. Hoffmann , C. Dreyer , I. Ramnarine , and A. Magurran . 2012. “Reasons for the Invasive Success of a Guppy ( *Poecilia reticulata* ) Population in Trinidad.” PLoS One 7, no. 5: e38404. 22693621 10.1371/journal.pone.0038404 PMC3365015

[ece370813-bib-0072] Smith, B. R. , and D. T. Blumstein . 2008. “Fitness Consequences of Personality: A Meta‐Analysis.” Behavioral Ecology 19: 448–455.

[ece370813-bib-0073] Soto‐Galera, E. , J. Paulo‐Maya , E. López‐López , J. A. Serna‐Hernández , and J. Lyons . 1999. “Change in Fish Fauna as Indication of Aquatic Ecosystem Condition in Río Grande de Morelia–Lago de Cuitzeo Basin, Mexico.” Environmental Management 24: 133–140. 10341069 10.1007/s002679900221

[ece370813-bib-0074] Swain, D. P. 2011. “Life‐History Evolution and Elevated Natural Mortality in a Population of Atlantic Cod ( *Gadus morhua* ).” Evolutionary Applications 4: 18–29. 25567950 10.1111/j.1752-4571.2010.00128.x PMC3352523

[ece370813-bib-0075] Therneau, T. M. , P. M. Grambsch , T. M. Therneau , and P. M. Grambsch . 2000. The Cox Model. New York: Springer.

[ece370813-bib-0076] Tsutsui, N. D. , A. V. Suarez , and R. K. Grosberg . 2003. “Genetic Diversity, Asymmetrical Aggression, and Recognition in a Widespread Invasive Species.” Proceedings of the National Academy of Sciences 100: 1078–1083. 10.1073/pnas.0234412100 PMC298729 12538869

[ece370813-bib-0077] Urban, M. C. , G. Bocedi , A. P. Hendry , et al. 2016. “Improving the Forecast for Biodiversity Under Climate Change.” Science 353: aad8466. 27609898 10.1126/science.aad8466

[ece370813-bib-0078] Valero, A. , C. Macías Garcia , and A. E. Magurran . 2008. “Heterospecific Harassment of Native Endangered Fishes by Invasive Guppies in Mexico.” Biology Letters 4: 149–152. 18211863 10.1098/rsbl.2007.0604 PMC2429932

[ece370813-bib-0079] Vilizzi, L. , G. H. Copp , J. E. Hill , et al. 2021. “A Global‐Scale Screening of Non‐Native Aquatic Organisms to Identify Potentially Invasive Species Under Current and Future Climate Conditions.” Science of the Total Environment 788: 147868. 34134389 10.1016/j.scitotenv.2021.147868

[ece370813-bib-0080] Vimercati, G. , S. Kumschick , A. F. Probert , L. Volery , and S. Bacher . 2020. “The Importance of Assessing Positive and Beneficial Impacts of Alien Species.” NeoBiota 62: 525–545.

[ece370813-bib-0081] Walker, S. , A. Monks , and J. Innes . 2019. “Thermal Squeeze Will Exacerbate Declines in New Zealand's Endemic Forest Birds.” Biological Conservation 237: 166–174.

[ece370813-bib-0082] Wohl, E. , J. Castro , B. Cluer , et al. 2021. “Rediscovering, Reevaluating, and Restoring Lost River‐Wetland Corridors.” Frontiers in Earth Science 9: 653623.

[ece370813-bib-0083] Zhao, D. , and P. Feng . 2015. “Temperature Increase Impacts Personality Traits in Aquatic Non‐Native Species: Implications for Biological Invasion Under Climate Change.” Current Zoology 61, no. 6: 966–971. 32256532 10.1093/czoolo/61.6.966 PMC7098674

